# Editorial: The Social Nature of Emotions

**DOI:** 10.3389/fpsyg.2016.00896

**Published:** 2016-06-14

**Authors:** Gerben A. van Kleef, Arik Cheshin, Agneta H. Fischer, Iris K. Schneider

**Affiliations:** ^1^Department of Social Psychology, University of AmsterdamAmsterdam, Netherlands; ^2^Department of Human Services, University of HaifaHaifa, Israel; ^3^Department of Psychology, VU University AmsterdamAmsterdam, Netherlands

**Keywords:** emotion, emotional expression, emotion processing, group processes, culture, social interaction, affective science

Emotions are a defining aspect of the human condition. They pervade our social and professional lives, influence our thinking and behavior, and profoundly shape our relationships and social interactions. Traditionally, emotions have been conceptualized and studied primarily as individual phenomena, with research focusing on cognitive and expressive components and on physiological and neurological processes underlying emotional reactions. Over the last two decades, however, an increasing scholarly awareness has emerged that emotions are inherently *social*—that is, they tend to be elicited by other people, expressed toward other people, and regulated to influence other people or to comply with social norms (Parkinson, 1996; Van Kleef, 2009; Fischer and Manstead, in press). Despite this increasing awareness, the inclusion of the social dimension as a fundamental element in emotion research is still in its infancy (Fischer and Van Kleef, 2010). To stimulate further theorizing and research in this area, the current research topic brings together the latest cutting-edge research on the social nature of emotions.

A growing literature supports the notion that emotions are tightly weaved into the fabric of our social lives (for a comprehensive review, see Van Kleef, 2016). For instance, research has demonstrated that social-contextual influences (e.g., norms, group membership) systematically shape the experience, regulation, and expression of emotions (e.g., Doosje et al., 1998; Clark et al., 2004; Fischer and Evers, 2011). Other studies have begun to uncover how social factors such as power differentials and culture influence the recognition and interpretation of emotional expressions (e.g., Elfenbein and Ambady, 2003; Mesquita and Markus, 2004; Stamkou et al., 2016). Still other work has documented how (behavioral) responses to the emotional expressions of other people are shaped by the social context, for instance in close relationships (e.g., Clark and Taraban, 1991; Guerrero et al., 2008), group settings (e.g., Barsade, 2002; Cheshin et al., 2011; Heerdink et al., 2013), conflict and negotiation (Van Kleef et al., 2008; Adam et al., 2010), customer service (Staw et al., 1994; Grandey et al., 2010), and leadership (Sy et al., 2005; Van Kleef et al., 2010b).

The social nature of emotions can be meaningfully analyzed at four different levels of analysis: the individual, dyadic, group, and cultural level (Keltner and Haidt, 1999). Even though these levels of analysis are not always mutually exclusive and some studies can be situated at multiple levels, this typology affords a useful organizing principle for discussing the contributions to the current research topic, which shed new light on the social nature of emotions at each of these levels.

## Individual level

At the individual level of analysis, critical research questions are how the social context influences the experience, regulation, and expression of emotions. Even when analyzed at the individual level of analysis, the deeply social nature of many emotions is apparent. An example of such an emotion is gratitude, which tends to arise in the context of social interactions with benevolent others and has been found to benefit mental health (Emmons and McCullough, 2003) as well as the quality of interpersonal relationships (Algoe et al., 2008; Lambert et al., 2010). As part of the current research topic, Fox et al. conducted one of the first studies into the neural underpinnings of this deeply social emotion. They found that ratings of gratitude correlated with brain activity in the anterior cingulate cortex and the medial prefrontal cortex, thus illuminating the neural networks that are activated when individuals are confronted with the goodwill of others.

Whereas the experience of gratitude implies that one is positively disposed toward another person, the experience of negative emotions such as schadenfreude (when observing the misfortune of another person) and gloating (when causing another's misfortune) imply a negative relationship. Even though schadenfreude and gloating are both associated with the adversity of others, Leach et al. argue that the two emotions can have very different consequences. They demonstrate in two studies that schadenfreude and gloating are distinct in terms of their associated situational features, appraisals, experience, and expression. These findings add nuance to the literature on (malicious) pleasure and begin to uncover the differential social roles that are played by schadenfreude and gloating.

Another inherently social emotion, nostalgia, involves a fond recollection of people and events in the past. Growing evidence indicates that nostalgia increases positive affect and decreases negative affect (Sedikides et al., 2008). Drawing on the social dimension of nostalgia, Cavanagh et al. argued and demonstrated that the alleviating effect of nostalgia on feelings of sadness depends on a person's social connectedness. Specifically, they found that memories of nostalgic vs. ordinary events influenced recovery from a sad mood depending on attachment insecurity, such that participants with low insecurity benefited from nostalgia whereas people with high insecurity did not. These findings indicate that the benefits of nostalgia depend on confidence in the quality of one's social relationships, further attesting to the intrinsically social constitution of the emotion.

Whereas some emotions (such as the ones discussed above) are almost necessarily social, other emotional responses may be elicited by social as well as non-social events. For instance, emotions such as pride or happiness may arise when non-social goals are achieved (e.g., succeeding at an exam), but they can also be elicited by the social evaluations of other people (e.g., praise). Wiggert et al. argued that emotional responses to social evaluations by others are modulated by gender. They found that positive evaluations expressed by men elicited stronger facial electromyography responses in both genders, whereas arousal was higher when positive evaluations were expressed by the opposite gender. These results suggest that emotionally evocative processes unfold differently depending on the gender of the interaction partners.

Despite the fact that research on the social nature of emotion is blossoming, developmental studies are relatively scarce. Extending classic work by Fridlund (1991) on the potentiating effects of the (implicit) presence of an audience on smiling, Visser et al. investigated the influence of the co-presence of a peer on children's expressions of happiness after winning a large first prize or a small consolation prize in a competitive game. They found that co-presence positively affected children's happiness only when receiving the first prize. Children who received the first prize were perceived as happier when they were in the presence of a peer who received the consolation prize than when they were alone. Conversely, children who received the consolation prize were rated as equally (un)happy when they were alone or in the presence of a peer. This indicates that children's smiling, much like adults' smiling, is influenced by the social context.

## Dyadic level

At the dyadic level of analysis, dominant research themes revolve around how people perceive, interpret, and respond to the emotional expressions of interaction partners, and how such effects are shaped by the social context. One way in which emotional expressions influence observers is by proving information about the expresser's interpretation of a situation (Manstead and Fischer, 2001; Van Kleef, 2009). Integrating theorizing on attribution, appraisal processes, and the use of emotions as social information (EASI), the contribution by Van Doorn et al. examined how emotional expressions influence attributions of agency and responsibility under conditions of ambiguity. Across three studies, they found that expressions of regret fueled inferences that the expresser was responsible for an adverse situation, whereas expressions of anger signaled that someone else was responsible. These results show that emotional expressions can help people make sense of ambiguous social situations by informing attributions that correspond with the appraisal structures that are associated with discrete emotions.

In a more applied vein, Cheshin et al. examined how people use the emotional expressions of professional baseball pitchers to make predictions regarding their upcoming pitches. Participants in their study expected pitchers with happy expressions to throw more accurate balls, pitchers with angry expressions to throw faster and more difficult balls, and pitchers with worried expressions to throw slower and less accurate balls. Participants also expected that batters would be more likely to swing at balls thrown by pitchers showing happy facial expressions, and these predictions were marginally associated with batters' actual swinging. These findings provide first-time evidence that the information that is conveyed by emotional expressions can potentially be leveraged to enhance performance in professional sports.

A considerable body of research indicates that social decision making is strongly influenced by the emotional expressions of interaction partners (Van Kleef et al., 2010a). However, the majority of this work has been conducted with healthy participants. de la Asuncion et al. investigated responses to emotional expressions of interaction partners in the context of fair vs. unfair decisions among healthy individuals and patients with schizophrenia. They found that healthy participants' behavioral responses to their interaction partners' unfair decisions were influenced by the partners' emotional expressions, whereas schizophrenia patients' decisions were not affected by the proposers' emotions. This finding indicates that schizophrenia patients have specific problems with processing and integrating emotional information, which jeopardizes the quality of their social relationships.

When a social relationship is threatened by a transgression, an apology by the offender can help to restore the relationship by eliciting forgiveness. For instance, apologizing for an outburst of anger has been found to alleviate negative consequences of the anger expression for impressions and desire for future interaction (Van Kleef and De Dreu, 2010). Extending the literature on the social effects of apologies, Beyens et al. found that participants who were punished by an interaction partner after a failure reacted less aggressively when the partner apologized afterward than when the partner did not apologize. They further found that female (but not male) participants held enhanced implicit attitudes toward the apologizing opponent. These findings confirm that apologies can dampen reactive aggression after wrongdoing.

Shifting the temporal perspective, Niven et al. investigated the role of emotions in the process of building new relationships, examining whether attempts to improve others' feelings can help people make connections in social networks. Across two studies, they found that the use of interpersonal emotion regulation strategies predicted growth in popularity in work and non-work interactions, although different strategies of interpersonal emotion regulation had differential effects. Behavioral strategies (e.g., providing comfort or reassurance) were positively associated with popularity, while cognitive strategies (e.g., changing a person's appraisals about the situation) were negatively associated with popularity. These findings shed new light on the role of emotions in the formation of new relationships.

The social signaling function of emotions may be particularly critical in settings where individuals are confronted with potentially threatening or harmful stimuli. In such circumstances, expressions of fear or pain may serve an important warning function (Williams, 2002). Accordingly, conscious observation of others' painful facial expressions has been found to increase pain perception in observers and to facilitate behavioral response tendencies. Extending this line of research, Khatibi et al. observed that ratings of the painfulness of aversive stimulation were higher following subliminal presentation of painful as opposed to happy expressions. Furthermore, they found that participants' tendencies to respond faster to targets in a computer task that were preceded by aversive stimulation was especially pronounced when participants were presented with subliminal painful expressions. This study indicates that even subliminal exposure to painful expressions can increase pain perception and enhance behavioral response tendencies.

In a related vein, Khatibi et al. examined how individuals respond to ambiguous painful facial expressions as a function of how they think about pain—more specifically, whether individuals have a tendency to “catastrophize” pain experiences. The authors created ambiguous pain expressions by morphing facial expressions of pain with facial expressions of happiness. In an incidental learning task, high (but not low) pain catastrophizers responded faster to targets appearing at the location predicted by painful expressions than to targets at the location predicted by happy expressions, suggesting that high pain catastrophizers are more likely to interpret ambiguous facial expressions of pain in a negative, pain-related manner. This interpretation bias was mitigated when explicit cues of threat vs. safety were provided, corroborating the notion that emotional expressions are particularly informative in the absence of more direct sources of information (Van Kleef, 2016).

## Group level

At the group level of analysis, researchers study how emotional patterns in groups shape the evolution of group norms and goals, group cohesion, differentiation from other groups, and the behavior of individual group members, among other things. As part of the current research topic, Delvaux et al. investigated in three studies how group members' emotional fit with their group is associated with their level of identification with the group. A cross-sectional study and two longitudinal studies point to a positive and bidirectional association between group identification and emotional fit, such that group identification and emotional fit either mutually reinforce or mutually dampen each other over time. This finding sheds new light on the temporal emotional dynamics of group identification.

Group identification tends to develop more readily in groups of physically co-located individuals than in groups of individuals who are situated at different locations and who are communicating via computer-mediated technology. Järvelä et al. investigated how communicating via such technologies influences the synchronization of physiological activity across individuals, which has been proposed as an underlying mechanism of emotional contagion and resultant feelings of similarity and identification (Hatfield et al., 1993; Hess and Fischer, 2016). A text chat option provided intermittent communicative emotional expressions to the group, while heart rate visualization showed continuous information about each group member's physiological state and their dyadic linkage to other group members. The opportunity of text chat increased heart rate synchrony regardless of physical presence, whereas heart rate visualization only increased synchrony within non-co-located dyads. Järvelä and colleagues speculated that emotional contagion is a more natural pathway to interpersonal synchrony in physically co-located groups, which reduces the perceived informational value of physiological information about other group members.

When it comes to using emotional information from fellow group members to make sense of situations, a relevant question is how the emotional expressions of group members are combined. One type of information that may be gleaned from emotional expressions in groups is whether one's behavior is deemed acceptable, with expressions of happiness signaling acceptance and expressions of anger signaling rejection (Heerdink et al., 2013). Heerdink et al. examined how many members of a group need to express their anger to influence a deviant group member. In two studies, they found that each additional angry reaction linearly increased the extent to which a deviant individual felt rejected. This felt rejection was found to promote conformity to the group norm when the deviant was motivated to seek reacceptance in the group and the shift toward conformity could be observed by the group. These findings highlight how emotional expressions may act in the interest of group goals by informing members about the desirability of their behavior.

Taking an intergroup approach, Furley et al. investigated responses to emotional expressions by teammates vs. opponents. Drawing on EASI theory (Van Kleef, 2009, 2016), Furley and colleagues argued and showed that emotional expressions take on different meanings and invite differential responses depending on whether they are emitted by members of one's own group or a competing outgroup. In particular, they found that pride expressions by opponents inspired negative emotions and cognitions and pessimistic expectancies regarding the performance of one's own team, whereas pride expressions by teammates instilled more positive emotions, cognitions, and performance expectations. These findings emphasize the importance of the social context in shaping the interpretation of emotional expressions.

## Cultural level

At the cultural level of analysis, the challenge is to understand the emotional interface between the individual and his or her cultural surroundings, which includes cultural influences on the emotion process as well as the effects of cultural fit on emotional functioning. Culture-specific patterns of emotions reflect cultural values and priorities (Mesquita, 2003). Accordingly, individuals within a given culture tend to experience similar patterns of emotions when confronted with similar situations. As such, the extent to which an individual's emotions are similar to the culture's average emotional pattern in the situation reflects his or her adoption of cultural values and priorities. In their contribution to the current research topic, De Leersnyder et al. examined whether such “emotional fit with culture” is associated with psychological well-being. They measured emotional fit with culture by comparing respondents' emotional patterns to the average cultural pattern for the same type of situation, comparing individuals from Korea, Belgium, and the United States. The results revealed that psychological well-being was predicted by emotional fit with culture in autonomy-promoting situations at work in the United States, in relatedness-promoting situations at home in Korea, and in both autonomy-promoting and relatedness-promoting situations in Belgium. These findings suggest that the experience of culturally appropriate patterns of emotions contributes to psychological well-being.

The ability to show emotional or behavioral responses that fit with one's culture requires an awareness of prevailing cultural norms and values. Whenever, such norms are not apparent, people may infer them based on the emotional expressions of others (Hareli et al., 2013; Van Kleef, 2016). Hareli et al. examined cross-cultural differences in how individuals use group members' emotional expressions to learn group norms. Consistent with research at the group level of analysis (Heerdink et al., 2013; Heerdink et al.), across cultures anger expressions were perceived as a stronger signal of norm violations than were sad or neutral expressions. However, whereas people in Germany and Israel were better able to learn the norm based on expressions of anger, people in Greece were better able to learn the norm based on expressions of sadness. These results indicate that the interpersonal effects of emotional expressions vary across cultures, perhaps as a results of the differential appropriateness of certain emotional expressions in different cultural contexts (see Van Kleef, 2016).

## Conclusion

There is a growing scholarly awareness that emotions are intrinsically social in that they are typically elicited, expressed, regulated, perceived, interpreted, and responded to in social settings. It is clear from the articles in this research topic that the study of the social nature of emotions is blossoming. The contributions cover a wide range of exciting new questions that span the individual, dyadic, group, and cultural levels of analysis. However, research at the group and cultural levels of analysis is comparatively underrepresented. This is no doubt due to the fact that such research is often complicated and time-consuming to conduct. These difficulties notwithstanding, research at the group and cultural levels of analysis is critical for our understanding of the social nature of emotions, and we call for more research in these domains.

It is notable that the contributions to this research topic employed a rich variety of methodologies, including correlational, longitudinal, and experimental designs involving behavioral, self-report, cardiovascular, and neurological measures. To reach the next frontier in the study of the social nature of emotions, it will be important to incorporate multiple measures in our research designs so as to facilitate cross-validation and interpretation of findings. Such integration promises to further enhance understanding of how individuals process their own and others' emotions, and how they respond to these emotions as a function of the relational, group, or cultural context.

## Author contributions

GvK wrote the article; AC, AF, and IS provided comments on the draft.

## Funding

Preparation of this article was supported by a grant from the Netherlands Organisation for Scientific Research (NWO 452-09-010) awarded to the first author.

### Conflict of interest statement

The authors declare that the research was conducted in the absence of any commercial or financial relationships that could be construed as a potential conflict of interest.
